# Utility of large language models as information tools for nursing care in gout: a comparative study of DeepSeek and ChatGPT

**DOI:** 10.3389/fmed.2026.1758735

**Published:** 2026-05-07

**Authors:** Xia Pan, Yali Wang, QiaoLan Yang, Jing Wang, Yun Tong, Duanfeng Zhang, Xiaofeng Lv, Chun Zheng, Miaoyin Wu, Tianwang Li, Li Tang, Zhengping Huang

**Affiliations:** 1 Department of Rheumatology and Immunology, The Affiliated Guangdong Second Provincial GeneralHospital of Jinan University, Guangzhou, China; 2 Department of Hepatobiliary, Pancreatic and Hernia Surgery, The Affiliated Guangdong Second Provincial General Hospital of Jinan University, Guangzhou, China; 3 Department of Nursing, Sihui People's Hospital, Zhaoqing, China; 4 Department of Nursing, The Affiliated Guangdong Second Provincial General Hospital of Jinan University, Guangzhou, China; 5 Department of Rheumatology and Immunology, Sihui People's Hospital, Zhaoqing, China

**Keywords:** ChatGPT-4.0, DeepSeek-R1, gout, large language models (LLMs), nursing

## Abstract

**Background:**

With the rapid advancement of artificial intelligence, LLMs (LLMs) are now employed across diverse domains. In nursing, their capacity for high-quality content generation is especially promising, offering practical value for clinical management, research, and education. Among the leading Chinese models is DeepSeek-R1.

**Objective:**

This study aims to evaluate and compare the effectiveness of DeepSeek-R1 and ChatGPT-4.0 as online information sources for nursing professionals seeking evidence-based care strategies for gout patients.

**Methods:**

We identified the 15 highest-priority questions on gout and related nursing strategies by surveying the research site, patients, and healthcare providers. These questions, posed in Chinese, were separately submitted to DeepSeek-R1 and ChatGPT-4.0. The Flesch Kincaid Grade Level (FKGL) and the Flesch Reading Ease (FRE) were used to evaluate the readability of their answers. The mDISCERN score was employed to compare the accuracy of their responses, and the age of statistical reference materials was assessed to compare their timeliness. GraphPad Prism 8.0.1 was used for all statistical analyses and figure preparation.

**Results:**

Readability and citation characteristics differed between the two LLMs. The FKGL of DeepSeek-R1 (13.04 ± 1.62) exceeded that of ChatGPT-4.0 (11.41 ± 1.74; *p* = 0.013), whereas FRE was lower for DeepSeek-R1 (40.50 ± 8.12) than for ChatGPT-4.0 (49.08 ± 8.90; *p* = 0.010). The mDISCERN quality score was numerically higher for ChatGPT-4.0 (4.30 ± 0.73) than for DeepSeek-R1 (3.98 ± 0.70), but this difference was not statistically significant (*p* = 0.16). DeepSeek cited 21 sources and ChatGPT-4.0 23; clinical guidelines predominated in both corpora (38.1 vs. 47.8 %, respectively). The mean publication age (years elapsed from 2025) was significantly younger for DeepSeek-R1 (3.57 ± 2.33) than for ChatGPT-4.0 (5.42 ± 2.34; *p* < 0.05). In addition, DeepSeek-R1 provided 4 reference links were invalid.

**Conclusion:**

Both DeepSeek-R1 and ChatGPT-4.0 drew chiefly from high-level evidence and produced accurate, professional answers; ChatGPT-4.0 rendered them in markedly clearer prose. While DeepSeek-R1 offered more up-to-date citations, several of its reference links were non-functional.

## Introduction

The emergence and subsequent development of large language models (LLMs) such as ChatGPT-4.0 and DeepSeek have dominated the global technological discourse. These models represent the new frontier of artificial intelligence, utilizing machine learning techniques to process and generate language, thereby providing users with acceptable and understandable suggestions (1). The rapid development and widespread influence of LLMs have become a global phenomenon, sparking intense discussions about their potential applications and impacts in the medical field (2–6). In China, DeepSeek has garnered significant attention since its launch due to its ability to process massive amounts of data and generate coherent, user-friendly responses in a conversational format (5, 6). As of March 9, 2025, over 300 hospitals in China have adopted DeepSeek-R1, integrating LLMs into real-world clinical applications and hospital management-related tasks, primarily including clinical diagnosis and decision support, patient education, conducting scientific research, and hospital management systems, playing a positive role (5). However, a study on central nervous system tumors showed that different language models performed poorly in terms of the accuracy of treatment recommendations (6).

LLMs are mainly applied in the fields of disease health education, disease management, nursing training, and post-operative education (7–13). DosSantos et al. used ChatGPT to generate care plans for elderly lung cancer patients, finding that the care plans generated by ChatGPT were very similar in scope and nature to standard plans, demonstrating its potential value as a tool for optimizing cancer care decision support (12). Abnoosian K et al. developed a big data platform for self-monitoring follow-up of valvular disease patients in the Sichuan North region, with its core component being an online diagnostic and treatment system powered by ChatGPT. Ruksakulpiwat et al.'s research found that the medical information provided by ChatGPT can facilitate communication between patients and healthcare providers, enhance patients' self-care capabilities, and improve treatment outcomes (13). However, nearly all studies have only described the application efficacy of the large language model ChatGPT in nursing, with few comparative studies on DeepSeek-R1 and ChatGPT. As a Chinese open-source large language model, DeepSeek is widely used in daily life due to its convenience.

Gout is a common chronic disease, with a global prevalence that has been increasing year by year, and the age of onset gradually becoming lower. Gout attacks are associated with an increased risk of cardiovascular diseases and can also cause kidney damage and other metabolic syndromes (14–17). Globally, the initiation and continuation rates of uric acid-lowering therapy are very low, resulting in few patients achieving the serum uric acid target levels for uric acid-lowering therapy. Nurse-led care strategies are crucial for effectively improving gout treatment outcomes and enhancing patient disease outcomes (18, 19). Nurse-led care strategies primarily achieve this through comprehensive disease management, enhancing patients' disease awareness, thereby modifying their health behaviors, improving treatment adherence, and achieving standardized treatment goals. However, current overall disease awareness among gout patients remains relatively low, with primary sources of disease knowledge including the internet, social media, and family/friends (20). A qualitative study by Hao X et al. on gout patients revealed issues such as a lack of disease-related health knowledge among recurrent gout patients (21).

During the disease process, patients may choose to independently search for health information online due to healthcare providers not informing them in a timely manner, patients forgetting, or being unable to understand the information provided by healthcare professionals. With the emergence of LLMs, an increasing number of patients are using large language model tools such as DeepSeek-R1 and ChatGPT for health information queries. In fact, both healthcare professionals and patients may use large language model tools for medical information retrieval in the healthcare field. However, since the current data sources for LLMs are not professional medical data or clinical data, these models may sometimes generate false information that aligns with linguistic logic, a phenomenon known as “model hallucination,” which could directly harm patients if used without caution (22).

Therefore, we conducted a brief assessment of the utility of LLMs DeepSeek-R1 and ChatGPT in gout care strategies, with primary evaluation criteria including the readability, source, accuracy and timeliness of responses to common care questions for gout patients.

## Methods

### Proposing nursing problems

Research was approved by the Ethics Committee of Guangdong Second Provincial General Hospital (2024-KY-KZ-285-01). Written informed consent was obtained from all participants before any samples were collected. We collected questions related to gout nursing care from online search platforms, gout patients, and nursing staff. Ten gout patients were selected through purposive sampling. The course of their disease was (9.8 ± 6.454) years, among whom 2 had renal insufficiency and 4 received needle-knife therapy. All 10 patients experienced joint pain, and 8 developed gouty tophi. Fifteen nurses from the Department of Rheumatology and Immunology were invited to provide gout care issues for the study. The nurses had an average working experience of (6.4 ± 5.767) years and were all capable of providing health education. In the end, 45 questions were obtained. Two researchers conducted open coding on these 45 questions, extracted keywords, and categorized and summarized them. When a conflict of opinion arose between the two researchers, a third researcher intervened to make a decision. Finally, 15 of the most pressing questions were selected from the pool. For convenience in subsequent readability analysis, we translated the questions from Chinese into English. First, a bilingual translator with relevant medical background and another bilingual translator proficient in English independently translated the questions into English, producing drafts E1 and E2. A researcher from our team then independently integrated E1 and E2 to form E3. Next, E3 was back-translated by two bilingual translators who had not previously seen the original checklist, producing C1 and C2. After team discussion and revisions, a back-translated version C3 was created. By combining the original document, E3, and C3, E3 was optimized through multiple discussions to form the final English version of the checklist.

### DeepSeek-R1 and ChatGPT-4.0 operation

DeepSeek uses the R1 model, which is an open-source model and can be used for free. Accessed via the official web platform (https://www.deepseek.com) using a standard web browser (Google Chrome 126.0.6478.126). For each of the 15 questions, we posed a question to DeepSeek-R1, requiring it to provide data sources when answering. We recorded each response and manually searched the sources of the answers, then statistically analyzed the sources.

Chat GPT uses version 4.0, only utilizes basic functions. Accessed via the OpenAI official API using Python 3.9.7, with the “openai” Python package (version 1.35.0). For each of the 15 questions, ChatGPT was prompted to provide answers, with the content of each response recorded. The sources of data provided by ChatGPT during the response process were statistically analyzed.

Both models were applied with a unified system prompt (see the full prompt in Response to Comment 5), with no additional role specification (i.e., the models were not assigned specific roles such as “nurse” or “doctor”). The parameter settings were as follows: temperature was set to 0.7 (the default for both models, balancing creativity and consistency), top_p was set to 0.95 (the default for both models, regulating output diversity), and maximum output length was limited to 2,048 tokens, which was sufficient for generating detailed responses to the nursing questions. Stochastic sampling was enabled as the default setting for both models. Internet browsing was activated for both models: DeepSeek-R1 used the built-in “DeepSeek Browser” plugin, while ChatGPT-4.0 used the “Browse with Bing” plugin. Citation tools were enabled for both models via their built-in citation generation functions, with no extra plugins required. No other third-party plugins were used for either model.

### A comprehensive overview of the prompts used in DeepSeek-R1 and ChatGPT

Please provide evidence-based answers to the following question about gout nursing care. The content should focus on clinical practice and align with current mainstream medical guidelines (e.g., 2021 ACR/EULAR gout guidelines, Chinese Guidelines for the Diagnosis and Treatment of Gout 2023). Each answer must clearly indicate its data sources (including the title, author, publication year, and journal of the reference). The language should be concise and rigorous, avoiding ambiguous expressions. If there are conflicting viewpoints in the literature, please present them objectively. Please do not use overly popular language; ensure the professionalism of the content while maintaining readability for nursing professionals.

### Analysis of the content of the answers

All queries were submitted between 08:00 and 12:00 (GMT+8) on 20 August 2025. Both models were run with default temperature settings, browsing plugins enabled, and an identical, fixed prompt: “[prompt content].”

### Readability measurement

Readability evaluation assesses the level of reading comprehension required for readers to understand written materials, used to evaluate the ease or difficulty of understanding textual materials. It is primarily assessed by calculating the average sentence length and the average number of syllables per word (23). This study used the online application Readable (https://readable.com, Added Bytes Ltd., Brighton, England) to evaluate the readability of the text generated by DeepSeek-R1 and ChatGPT-4.0. FRE and FKGL were used to evaluate text readability, as these methods are commonly used to assess the readability of internet-based health science popularization texts (24). FRE score ranges from 0 to 100, with lower scores indicating higher reading difficulty (25). The FKGL score ranges from 0 to 18, indicating the number of years of education required to understand the text, with lower scores indicating better readability (26).

### Accuracy measurement

Related studies show that large language models can help patients enhance their self-management capabilities, but it is necessary to evaluate the accuracy of the content provided by these models (27). Therefore, one chief physician and one chief nurse from the Department of Rheumatology and Immunology were invited to participate, both of whom have more than 10 years of clinical experience in rheumatology and immunology. The two experts evaluated the accuracy of answers generated by different language models. Prior to evaluation, the two texts were de-identified. The two experts used the mDISCERN tool (28) to evaluate the professionalism of the response texts generated by the two language models. The mDISCERN tool is a modified version of the DISCERN tool, designed to assess the quality of written consumer health information regarding treatment options. The meanings of mDISCERN scores are as follows: 1 or 2 points indicate no, low, or significant defects; 3 points indicate partial, moderate, or potentially important but not significant defects; 4 or 5 points indicate high or extremely low defects. To avoid bias, we provided training on the scoring criteria to the healthcare professionals involved in the evaluation prior to scoring.

### Source of information

Large language models (LLMs), such as ChatGPT-4.0 and DeepSeek-R1, offer advanced natural language processing capabilities, but they also raise concerns regarding the accuracy of medical applications, including potential inherent biases, hallucinations, and output reliability issues (29, 30). Moreover, AI-generated false or distorted information often becomes more persuasive during dissemination, which can exacerbate cybersecurity and online fraud issues. Therefore, assessing the sources of medical information generated by large language models is particularly crucial for medical safety. This study manually searched the information sources of the data materials provided during the response process to ensure the authenticity of the data materials. Additionally, the information sources of the data materials were statistically analyzed to further validate the professionalism of the responses. The information sources were categorized into the following types: clinical guidelines, SRMAs, original research, monographs, case reports, and social media.

### Timeliness

By searching the reference materials in DeepSeek-R1 and ChatGPT-4.0, and eliminating false information, we derived the authentic reference materials and recorded the time of their creation. Given that this study was conducted in 2025, we calculated the time difference between the reference materials and the year 2025. A smaller time gap means the sources are more recent and up-to-date.

### Statistical analysis

GraphPad Prism 8.0.1 was used for all statistical analyses and figure preparation. Continuous variables are expressed as mean ± standard deviation. Between-group comparisons were performed with unpaired *t*-tests or Mann–Whitney *U*-tests, as appropriate. A two-sided *P* < 0.05 was considered statistically significant unless otherwise stated.

## Results

### Question identification

**Figure 1:**
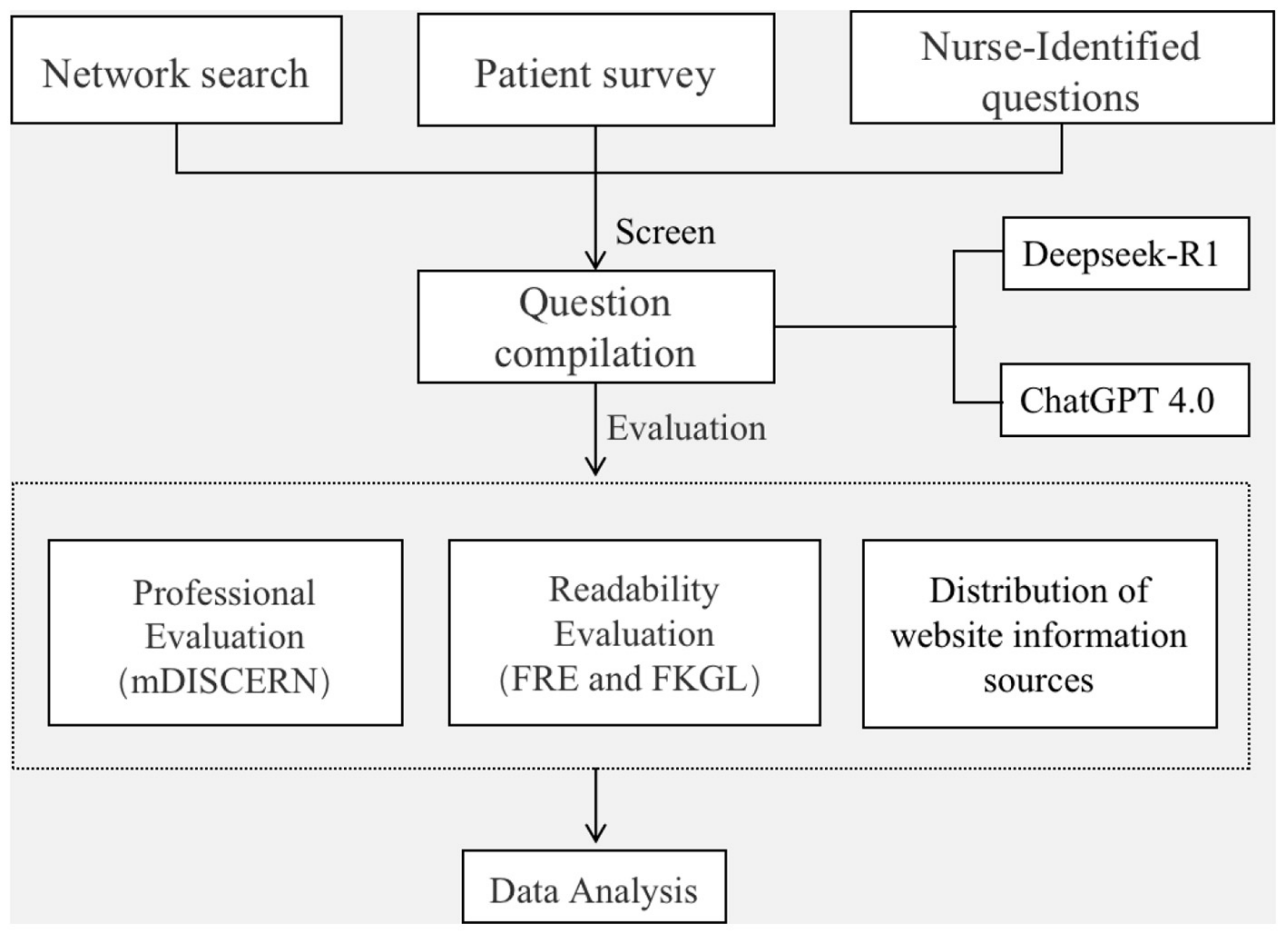
Flowchart of question screening and evaluation.

According to the flowchart of question screening (Figure 1), we identified 15 key questions related to gout care strategies. These questions cover seven main areas: dietary management, pain management, wound care, medication management, health education, Non-pharmacological treatment modalities, and the identification of complications (Table 1).

**Table 1:** Classification and content of the questions.

Classification	Content of the question
Dietary management	1) How do I develop a personalized, specific and implementable dietary guidance program?
	2) How do I manage protein supplementation and safe weight loss?
	3) How do I drink the correct amount of water, including how much, how often, and what kind of water to drink?
Pain management	4) What is the effective pain management when the conventional pain medications are not effective?
Wound care	5) How should I care at home for ulcerations over tophi?
Medication management	6) As a patient with impaired renal function, do I need to adjust the dosage of my uric acid-lowering medication?
	7) Do I need to continue taking uric acid-lowering medication after my uric acid level has returned to normal?
	8) When is the best time to start uric acid-lowering treatment?
Health education	9) How to design health education activities that are relevant to the needs of patients based on their age?
	10) What is the optimal frequency for monitoring uric acid?
	11) How to manage chronic gouty arthritis?
	12) How to assess the risk of gout attacks?
Non-pharmacological treatment modalities	13) How do I change the dressing for the wound after being discharged following tophus removal surgery?
	14) Are acupuncture and other Chinese medicine tactics tools applicable during acute gout attacks?
Complication recognition	15) How to recognize early manifestations of gout complications and manage patients with comorbid cardiovascular disease in a comprehensive manner?

### Readability analysis

**Figure 2:**
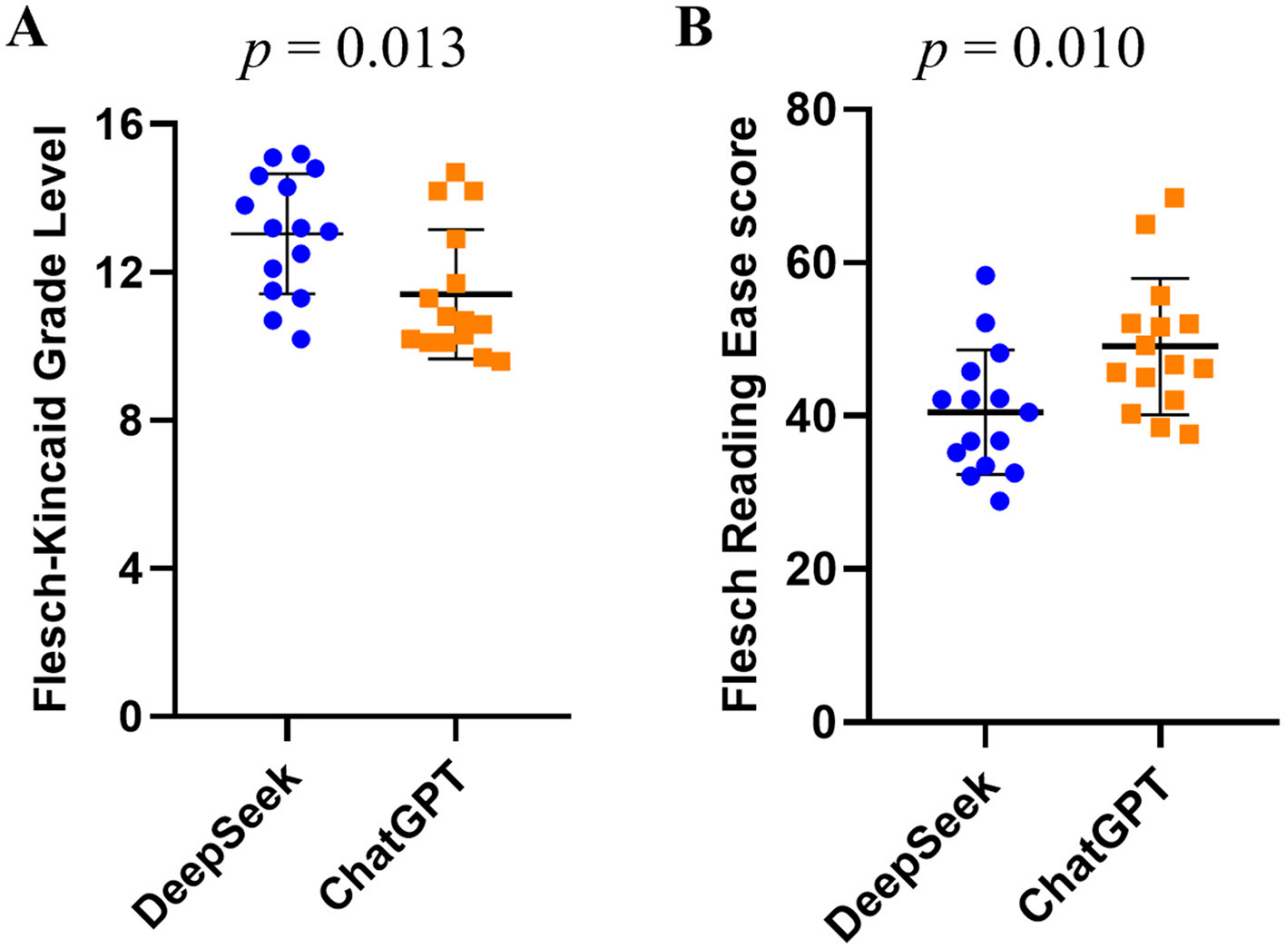
Evaluation of Readability: **(A)** Flesch Kincaid Grade Level and **(B)** Flesch Reading Ease Score for DeepSeek-R1 vs. ChatGPT-4.0.

In terms of readability, The FKGL scores for DeepSeek-R1 and ChatGPT-4.0 were 13.04 ±1.62 and 11.41 ± 1.74 (*p* = 0.013), respectively (Figure 2A). FRE scores for DeepSeek-R1 and ChatGPT-4.0 were 40.50 ± 8.12 and 49.08 ± 8.90 (*p* = 0.010), respectively (Figure 2B). These data indicate that ChatGPT-4.0 has significantly higher readability than DeepSeek-R1. However, the scores indicate that both texts have high reading difficulty, requiring readers to have reading proficiency at or above the high school graduate level.

### Accuracy analysis

**Figure 3:**
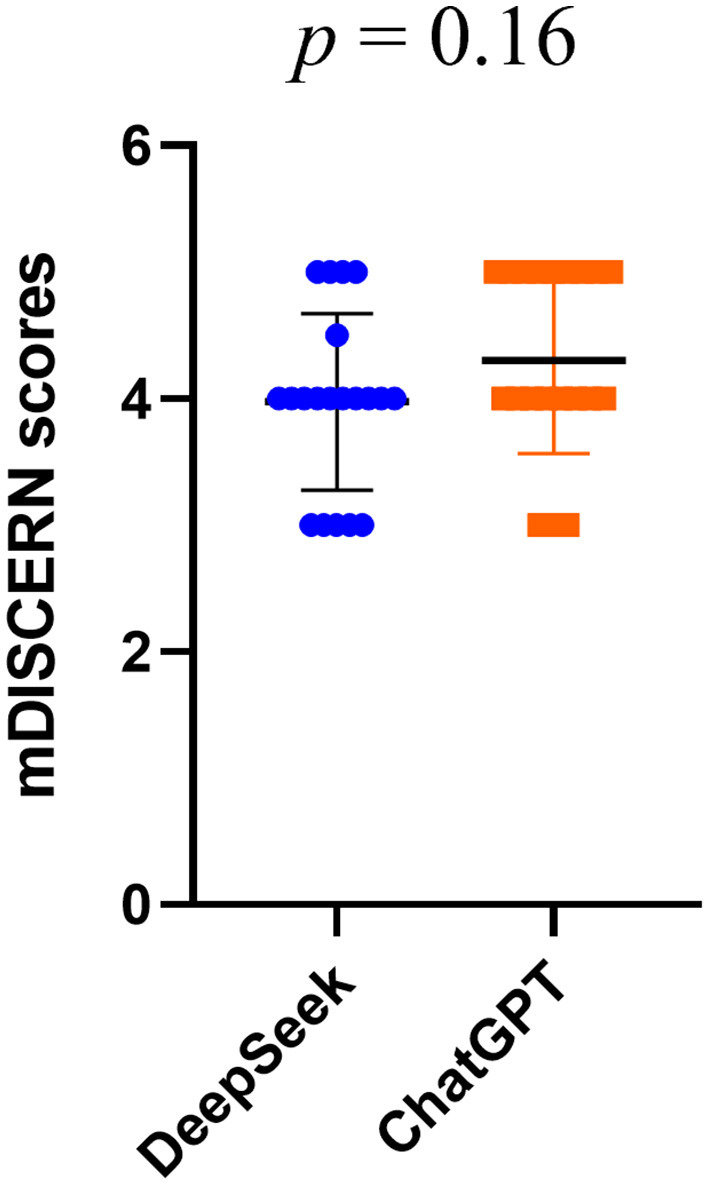
The mDISCERN scores for DeepSeek-R1 and ChatGPT-4.0.

The mDISCERN scores for DeepSeek-R1 and ChatGPT-4.0 were 3.98 ± 0.70 and 4.30 ± 0.73, respectively (*p* = 0.16) (Figure 3), indicating that both models produced content free of major flaws and with a reasonable level of accuracy. The mDISCERN inter-rater reliability was excellent, with an ICC of 0.84 (95 % CI 0.71–0.92).

### Information source analysis

**Figure 4:**
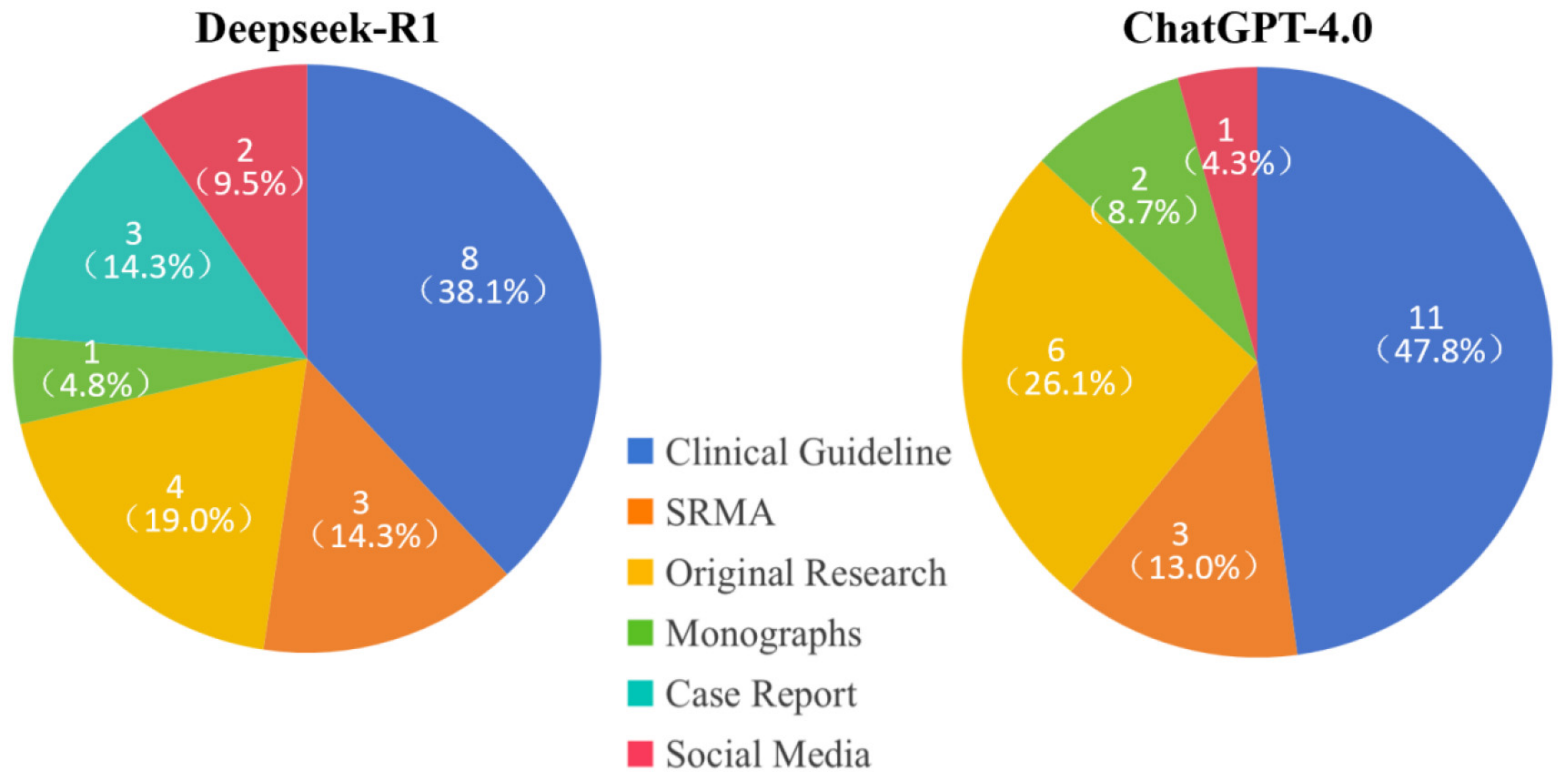
The reference source for DeepSeek-R1 and ChatGPT-4.0.

When answering identical clinical queries, DeepSeek and ChatGPT differed both in volume and in the hierarchy of evidence they cited (Figure 4). DeepSeek - R1 provided 25 papers, of which 4 contained false information, while ChatGPT provided 23. Clinical guidelines dominated both corpora, accounting for 38.1 % (8/21) of DeepSeek citations and 47.8 % (11/23) of ChatGPT citations. Original research comprised the next largest share: 19.0 % (4/21) for DeepSeek vs. 26.1 % (6/23) for ChatGPT. Systematic reviews/meta-analyses were cited at comparable frequencies (DeepSeek 14.3 %, ChatGPT 13.0 %). Monographs were rarely used (< 10 % in both sets). Notably, DeepSeek included case reports (14.3 %, 3/21) and twice as many social-media sources (9.5 %, 2/21) as ChatGPT (4.3 %, 1/23), whereas ChatGPT contained no case reports. Overall, ChatGPT concentrated its references on guidelines and original research, whereas DeepSeek exhibited a more even distribution across evidence tiers and incorporated a wider spectrum of publication types.

### Reference timeliness analysis

**Figure 5:**
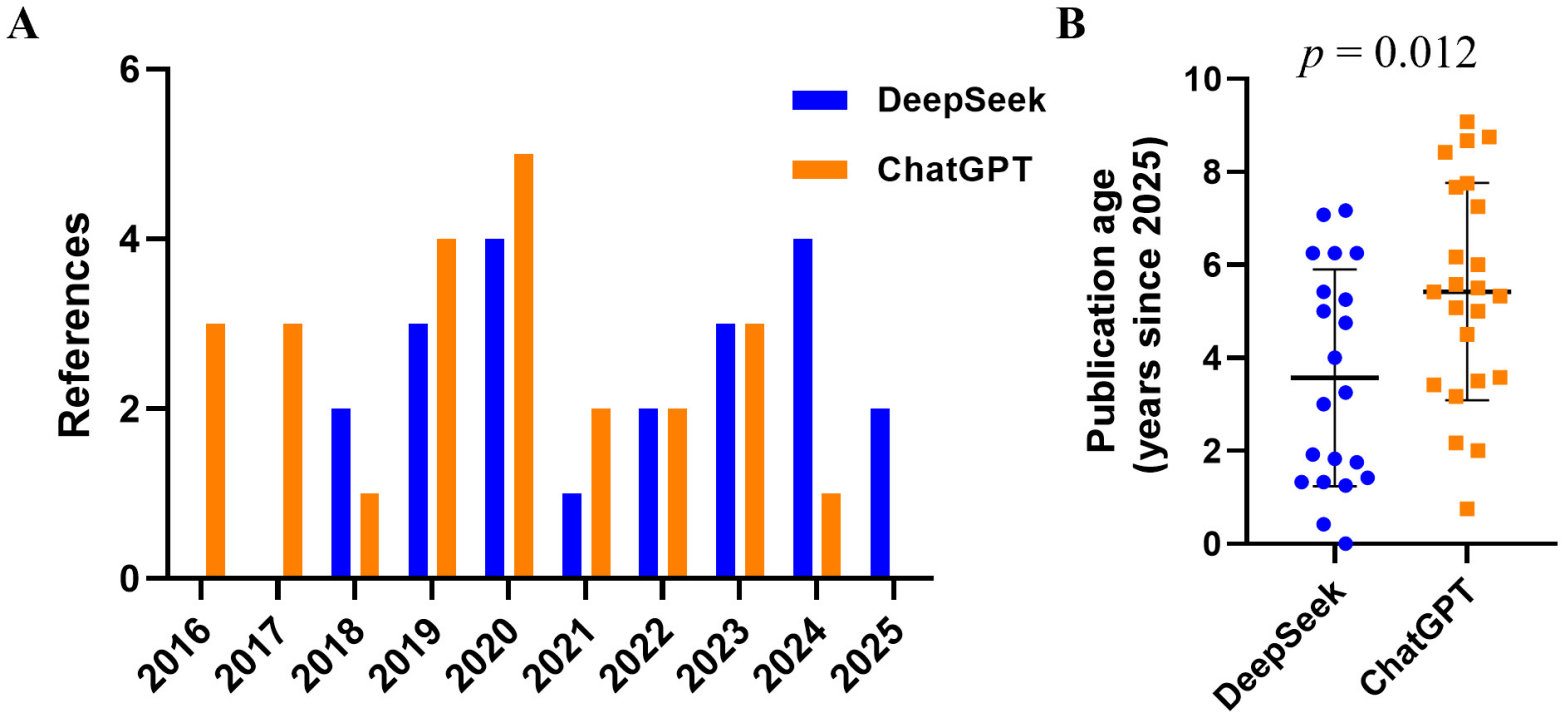
Analysis of reference timeliness. **(A)** The cited references across years. **(B)** The mean publication age since 2025.

The publication years of the citations retrieved by DeepSeek-R1and ChatGPT-4.0 are summarized in the Figure 5A. DeepSeek-R1supplied 21 references spanning 2018–2025, whereas ChatGPT-4.0 provided 23 references covering 2016–2024. Neither model showed a marked preference for very recent literature: the median publication year was 2020 for DeepSeek-R1 and 2019 for ChatGPT-4.0. DeepSeek-R1 exhibited a relatively even spread, with 3–4 citations per year between 2019 and 2020 and again in 2023–2024. ChatGPT-4.0 displayed a broader temporal range, including three citations from as early as 2016–2017, and peaked in 2020 (*n* = 5). Notably, ChatGPT-4.0 contained no 2025 items, whereas DeepSeek-R1 already included two 2025 publications. The mean publication age (years since 2025) for DeepSeek-R1 was 3.57 ± 2.33 years, compared with 5.42 ± 2.34 years for ChatGPT-4.0 (*p* = 0.012, Figure 5B), indicating that DeepSeek-R1 cites significantly more up-to-date references than ChatGPT-4.0. Taken together, both models relied predominantly on literature published within the last 8 years, with ChatGPT reaching slightly further into the past and DeepSeek showing earlier uptake of the most recent sources.

## Discussion

This study aimed to evaluate the utility of DeepSeek-R1 and ChatGPT-4.0 as online resources for users seeking gout care strategies. Low readability of large language model responses is a common phenomenon. The FKGL and FRE scores determine the reading level of an article based on the number of sentences and words, while LLMs often use long and complex sentences, excessive technical terms, and a wide range of training data sources, which result in low readability of their responses (31, 32). In this study, ChatGPT-40's answers are significantly more readable than DeepSeek-1's, with a statistically significant difference. DeepSeek- 1's responses have a high information density, with abundant data and details, using numerous subheadings, hierarchies, and abbreviations, requiring readers to have a certain level of medical background, making it suitable for doctors or nutritionists. ChatGPT-40's responses have moderate information density, with a simpler and more popular science-oriented language style compared to DeepSeek-R1, making it suitable for general readers. It also uses numerous tables and flowcharts to describe content, making the text clearer and easier to understand, suitable for patients, family members, and entry-level healthcare personnel. Both language models require users with a high school education or higher to fully comprehend the responses to questions posed during the research process. However, it is worth noting that both models organize certain content in their responses, presenting it in tables and flowcharts with clear logical structure, thereby reducing comprehension difficulty to some extent. However, the content of charts is not included in the calculation of FKGL and FRE scores. However, Chen LW et al.'s research indicates that if a defining action is added to the question (e.g., “I am a patient”), the language model typically provides answers suitable for individuals with a reading level below high school graduation (33). This suggests that LLMs can provide answers at different levels based on the asker's identity and cognitive level, indicating that LLMs can break down the professional-cultural barriers between medical knowledge and the general public, which is of great significance for reducing disparities in medical cognitive levels.

In terms of content, although the two models do not differ significantly in accuracy or professionalism, ChatGPT-4.0's answers cover every topic while also weaving in cultural context, psychological support and family involvement, delivering a truly person-centred, holistic vision of health management. DeepSeek-R1, while detailed in data and clear in structure, lacks dimensions such as cultural, psychological, and family considerations. Additionally, DeepSeek-R1 uses overly absolute language and fails to consider individual differences. For example, it suggests “2–4 cups of coffee per day to lower uric acid,” but does not mention individual differences (e.g., caffeine-sensitive individuals may experience arrhythmia). Therefore, this study suggests that ChatGPT-4.0 is better suited to equipping non-professionals with evidence-based, practical gout-care strategies and to helping them sift out online misinformation.

Our head-to-head comparison reveals two distinct citation philosophies. DeepSeek-R1 cited clinical guidelines, SRMAs, original research, monographs, case reports, and social media. ChatGPT-4.0 referenced clinical guidelines, SRMAs, original research, monographs, and social media, but no case reports. Clinical guidelines, SRMAs, and monographs are high-level evidence that ensure accuracy and professionalism, whereas case reports and social media present single-patient anecdotes or personal opinions that may reduce accuracy and professionalism. The proportions of high-level evidence (clinical guidelines, SRMAs, and monographs) were similar between DeepSeek-R1 and ChatGPT-4.0.

In addition, the study indicates that DeepSeek-R1 consistently taps more recent literature, potentially giving users the latest evidence-based developments in gout management. ChatGPT-4.0, while drawing from a larger and more heavily academic pool, relies on comparatively older material; whether this affects the clinical currency of its advice will depend on how rapidly the topic evolves. Future users may therefore consider DeepSeek-R1 when up-to-the-minute citations are paramount, whereas ChatGPT-4.0 remains valuable for its breadth, academic rigor and readability.

This study has certain limitations. Firstly, the scope of the study is limited to two widely used language models (DeepSeek-R1 and ChatGPT-4.0), as the present study focuses on exploring the practical value of these models for nursing professionals rather than conducting comprehensive benchmarking. While this pragmatic approach ensures that the findings are directly relevant to clinical end-users, it is acknowledged that excluding other major systems, such as models from Google or Anthropic, may limit the generalisability of the study's comparative results. Secondly, it primarily focuses on nursing management issues raised by the authors based on their experience with real patients. In practical application, however, the actual nursing needs of patients are more complex in clinical practice, and users' concerns are more diverse. Future research could expand the scope and depth of inquiries to gain a deeper understanding of the effectiveness of Deepseek-R1 and ChatGPT-4.0 in formulating gout care strategies. Thirdly, this study only examined the initial responses of Deepseek-R1 and ChatGPT-4.0 and did not investigate changes in their performance during interactions with users.

## Conclusion

By comparing the effectiveness of DeepSeek-R1 and ChatGPT-4.0 in providing gout care strategy information, this study found that both DeepSeek-R1 and ChatGPT-4.0 provided moderate quality medical knowledge, personalized care strategies, and supporting patient health education. DeepSeek-R1 leaned on newer citations, whereas ChatGPT-4.0 tapped a wider, overwhelmingly academic evidence base. Crucially, ChatGPT-4.0 rendered its content in clearer, more accessible language.

## Data Availability

The original contributions presented in the study are included in the article/supplementary material, further inquiries can be directed to the corresponding authors.
